# Designing Low-Cost Capacitive-Based Soil Moisture Sensor and Smart Monitoring Unit Operated by Solar Cells for Greenhouse Irrigation Management

**DOI:** 10.3390/s21165387

**Published:** 2021-08-09

**Authors:** Abdelaziz M. Okasha, Hasnaa G. Ibrahim, Adel H. Elmetwalli, Khaled Mohamed Khedher, Zaher Mundher Yaseen, Salah Elsayed

**Affiliations:** 1 Department of Agricultural Engineering, Faculty of Agriculture, Kafrelsheikh University, Kafr El-Sheikh 33516, Egypt; abdelaziz.okasha@agr.kfs.edu.eg (A.M.O.); hasnaa.gamil@agr.kfs.edu.eg (H.G.I.); 2 Department of Agricultural Engineering, Faculty of Agriculture, Tanta University, Tanta 31527, Egypt; adel.elmetwali@agr.tanta.edu.eg; 3 Department of Civil Engineering, College of Engineering, King Khalid University, Abha 61421, Saudi Arabia; kkhedher@kku.edu.sa; 4 Department of Civil Engineering, High Institute of Technological Studies, Mrezgua University Campus, Nabeul 8000, Tunisia; 5 New Era and Development in Civil Engineering Research Group, Scientific Research Center, Al-Ayen University, Thi-Qar 64001, Iraq; 6 College of Creative Design, Asia University, Taichung 41354, Taiwan; 7 Agricultural Engineering, Evaluation of Natural Resources Department, Environmental Studies and Research Institute, University of Sadat City, Minufiya 32897, Egypt; salah.emam@esri.usc.edu.eg

**Keywords:** cost-effective sensor, soil moisture, irrigation management, solar photo voltaic, monitoring and control

## Abstract

Precise and quick estimates of soil moisture content for the purpose of irrigation scheduling are fundamentally important. They can be accomplished through the continuous monitoring of moisture content in the root zone area, which can be accomplished through automatic soil moisture sensors. Commercial soil moisture sensors are still expensive to be used by famers, particularly in developing countries, such as Egypt. This research aimed to design and calibrate a locally manufactured low-cost soil moisture sensor attached to a smart monitoring unit operated by Solar Photo Voltaic Cells (SPVC). The designed sensor was evaluated on clay textured soils in both lab and controlled greenhouse environments. The calibration results demonstrated a strong correlation between sensor readings and soil volumetric water content (*θ_V_*). Higher soil moisture content was associated with decreased sensor output voltage with an average determination coefficient (*R*^2^) of 0.967 and a root-mean-square error (RMSE) of 0.014. A sensor-to-sensor variability test was performed yielding a 0.045 coefficient of variation. The results obtained from the real conditions demonstrated that the monitoring system for real-time sensing of soil moisture and environmental conditions inside the greenhouse could be a robust, accurate, and cost-effective tool for irrigation management.

## 1. Introduction

The development of Information Technology (IT) and the emergence of free and open-source technologies offer a suitable environment for scientific and technological innovations [1,2]. Over the last few decades, the utilization of advanced technology for agricultural purposes has been taken into consideration to control various agricultural practices and to make informed decisions based on the continuous monitoring of fields by employing different robust sensors [3,4]. Among the different agricultural practices, irrigation is the largest consumer of the available water budget worldwide, using more than 75% of freshwater resources [5,6]. In addition, it is expected that the problem will be getting worse in the forthcoming decades as a result of rapid population growth and expected climate change, which will lead to increased demands for freshwater [7]. In arid and semi-arid regions, agriculture plays a major role in the total national income and is considered a livelihood for most of the population, such as in Egypt [8]. However, being an arid region, Egypt faces a great challenge, and therefore irrigation is essential to provide necessary moisture for plant growth and development [9]. It is known that water scarcity is one of the biggest challenges and crises facing Egypt as a result of climate change, in addition to the construction of many development projects within the Nile basin countries (e.g., Ethiopia, Sudan) [10]. In this context, irrigation is generally based on the personal perspective of the farmer, and therefore there is an urgent need to change from traditionally used methods of monitoring irrigation scheduling that depend on a farmer’s experience to precision monitoring methods that are based on advanced scientific devices and advances in modern technologies that provide low-cost and effective solutions that improve different agricultural practices. Most growers will use modern technologies when they are affordable [11].

The accurate estimates of soil moisture can contribute to diverse engineering applications such as hydraulics, agronomy, and soil morphology physics [12,13]. Chemical, physical, biological, and mineralogical properties are significantly affected by soil moisture content [14]. Soil moisture content is a basic variable of the climate system, as it moderates the energy fluxes between the atmosphere and the land surface via its influence on evapotranspiration, and thus the monitoring of temporal and spatial variability of soil moisture content is important [15,16]. Soil is a vital element in any crop’s growth and development, and its main function is to keep and drain water, as well as to supply necessary nutrients to crops. Therefore, continuous monitoring of soil moisture content has received much attention in precision farming as a way to monitor crop water uptake in settings that rely on automated irrigation systems [17].

Several techniques and devices are used for measuring soil moisture content; they are divided into two categories: classical and modern techniques [18]. Classical techniques involve the thermo-gravimetric technique, which is known as the standard reference method for identifying soil moisture [19]. However, this method is laborious, destructive to the soil, and time consuming [20]. Another technique is the use of calcium carbide, which mainly depends on measuring the pressure of the produced gas resulting from the chemical reaction between soil moisture and calcium carbide [21]. Using appearance and feel method is another way to determine soil moisture, which mainly depends on the experience of farmer [22].

Modern techniques involve an assortment of sensors and devices, with the advantage of detecting changes in soil moisture before the crop reaches the stress stage [23]. Most of the adopted techniques include tensiometers that are used for determining matric potential. Finding the balance between the tension held by water in the soil particles and the negative pressure of water inside the device is the principle of this technique. Although tensiometers are easy to use, they have a low range of measurement [24]. Neutron scattering is another adopted method for measuring soil moisture [25]. The health risk associated with exposure to radiation emitted from the device is the major disadvantage of this technique [26,27]. Soil resistivity is another method for measuring soil moisture, which can be done through measuring either material resistivity that is in equilibrium with the soil or resistivity between two electrodes inserted into the soil. Although it is simple and low cost, it requires careful calibration and installation, and it is affected by soil salinity [28].

The common previous studies conducted on developing devices to measure soil moisture content are dielectric techniques featured for their accuracy, simple installation, and the wide range for measuring soil water content [29]. The concept behind employing the dielectric methods is based on the wide range between the dielectric constant or relative permittivity of air (ε_r_ ≈ 1) or solids soil (ε_r_ ≈ 2–5) and for pure water (ε_r_ ≈ 80) at room temperature. The dielectric constant of the soil is a function of water content [30]. While the operating principle of these techniques is based on the idea that a water molecule is a dipole due to the existence of two partial positive charges on the sides of the hydrogen atom, while on the oxygen atom there is a negative charge for the same molecule. Materials consisting of the molecules that are polarized permanently usually have great dielectric constants [31]. The dipolar nature of water molecules supply bulk water with matchless electrical properties that have long been the main target of measuring water in the soil [32].

Soil moisture sensors depending on the soil dielectric constant are classified by few methods according to their functionality, such as time domain reflectometer (TDR) [33], time domain transmissometry (TDT) [34], frequency domain refelectomerty (FDR) [35], and capacitance methods [36]. TDR basically relies on assessing the apparent soil permittivity by recording a pulsed electromagnetic signal’s travel time along a parallel waveguide [18]; however, it is still very expensive and has difficulties concurrent with the required waveform analysis, and thus developing alternative soil water content sensors is crucial when considering the total cost of agricultural operations [30]. Frequency and capacitance techniques offer a reliable option to TDR owing to the effective cost, monitoring continuity, data logging feasibility, and wide range of measurable soil types [37]. Generally, the soil water that exists between the sensor plates provides a noticeable significance in its capacitance, and thus the higher the water content and the greater the capacitance will be. This capacitance can then be measured electrically. However, other scholars such as Hanson and Peters [38] and Oates et al. [39] pointed out that different soil types may display dielectrically different properties.

The frequency domain (FD) established on capacitance of a capacitor is proportionate to the soil bulk permittivity, and it works at lower frequencies. When this capacitor fabricated from metal rods or plates, in parallel, is buried into the soil, the variations in the soil moisture produce changes in the operating frequency of the circuit. The permittivity of FD is fundamentally measured by determining capacitor charging time, whilst the FD switches the frequency of the oscillator within a certain range for recording the resonant frequency. In this frequency range the amplitude will be at maximum, representing the moisture in the soil surrounding those sensors [40,41]. In addition, counter to other techniques of soil moisture measurement such neutron scattering devices cover a wide measurement volume on effect [42]. These sensors are relatively short (small) and sensitive only to the immediate proximity surrounding them, and to ensure precise determination of soil moisture, more than one sensor should be installed. This not only enhances the sensor’s accuracy but also enables an increased sampling volume of changes in soil moisture [43].

Many studies showed that soil moisture sensor accuracy is influenced by various parameters, including soil physical properties; type, porosity and temperature [44,45]; and also chemical properties such as electrical conductivity (EC) and salinity [29]. Cardenas-Lailhacar and Dukes [46] tested three commercial soil moisture sensors under varying conditions of both temperature and salinity for irrigating landscapes and found two of them are sensitive to high salinity and/or high temperature, which makes their use more problematic in severe environments. Therefore, our research was based on the hypothesis that certain coating materials can eliminate the effect of salinity and temperature on soil moisture sensor readings.

Based on the forgoing literature, it seems clear that designing sensors with low-cost components and that consume less power will be useful. Based on those facts, the current research was initiated to propose a new material for coating soil moisture probes so as to eliminate the effect of soil temperature and salinity. Hence, the main objectives of the present study were to (i) design an inexpensive capacitive soil moisture sensor; (ii) calibrate the locally manufactured sensor in clay textured soils; and (iii) design and implement a smart monitoring unit for irrigation management in controlled greenhouse conditions.

## 2. Materials and Methods

The first stage of this research was a laboratory effort to design, test, manufacture, and calibrate a capacitive soil moisture sensor. In addition, the research involved designing a smart monitoring unit for sensing environmental conditions inside greenhouses. The second stage was to test the sensor in the field through real-time sensing of soil moisture content and detecting environmental parameters inside a greenhouse.

### 2.1. Design Stage of the Soil Moisture Sensor

The electrical design of the sensor consisted of three main circuits: an oscillation circuit, a low-pass filter, and a half-wave rectifier “peak detector circuit”. The electrical method used was based on using the soil moisture probe as a capacitor of passive low-pass filter. The capacitive soil moisture sensor circuit was designed and tested in the lab by Multisim program v.11.0.02 (Electronic Workbench group, Austin, TX, USA). During the lab tests, all electronic integrated-circuits were used in Dual In-line Package (DIP) type to be easily inserted in the breadboard.

#### 2.1.1. Oscillation Circuit

**Figure 1:**
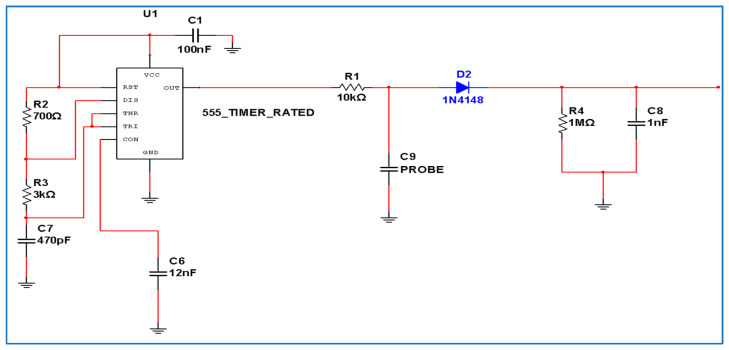
Schematic diagram of capacitive soil moisture sensor circuit.

An integrated circuit (IC) oscillator, the 555 IC, was selected to be used in a stable mode that outputs a square wave frequency of 430 kHz with an approximate duty cycle of 55%. This frequency was chosen to suit the maximum frequency that can be generated from the low cost 555 IC that was selected and to align with the cut-off frequency for the low-pass filter. The produced frequency and the duty cycle of the frequency wave were adjusted based on the following equations, which are available in the IC datasheet.

(1) $$Ton\;=\;0.693\;\left(Ra+Rb\right)\;C$$

(2) $$Toff\;=\;0.693\;\left(Rb\right)\;C$$

(3) $$F\;=\frac{1.44}{\left(Ra+2Rb\right)\;\times \;C}$$

(4) $$Duty\;cycle\;=\frac{\left(Ra+Rb\right)}{\left(Ra+2Rb\right)}\times 100$$

where Ton is the charging time, Toff is the discharging time, *F* is the output frequency, Ra is the Resistor linked between Vcc and discharge pin of 555, Rb is the resistor linked between discharge pin and connection point of trigger and threshold pins, and *C* is the capacitor linked between trigger with threshold and ground. Figure 1 shows the electric circuit of different components.

#### 2.1.2. Low-Pass Filter

The low-pass filter consisted of a resistor and a capacitor connected in series that was responsible for passing the low-frequency signal and attenuating the higher frequency signal. The output signal generated by the 555 oscillator is input to the *RC* low-pass filter via a 10 kΩ resistor connected to the output pin of the 555. The other end of the resistor was connected in series to one terminal of the capacitive sensor probe. The cut-off frequency was calculated according to this equation:

(5) $$Fc\;=\;\frac{1}{2\pi RC\;}$$

#### 2.1.3. Half-Wave Rectifier

The rectification circuit was used to change the alternating current (AC) to direct current (DC). The output triangle signal exits from the low-pass filter to half-wave rectification circuit (peak detector) consisting of 1N4148 diode, 100 nF, which is linked in series to produce a DC signal. To improve the signal and reduce the ripple, a 1 MΩ resistor was connected in parallel to the 100 nF capacitor, and the resistor and the capacitor were connected to the ground pole. Putting the diode in series prevents this capacitor from being fully discharged; when the signal goes low it will slow down to a certain point at which this diode stops conducting and becomes isolated and just the 1 MΩ resistor discharges the capacitor. Because it is no longer being refreshed by triangle waves, this 1 MΩ resistor with the 100 nF capacitor will have a time constant of 0.1.

#### 2.1.4. Sensor Probe

**Figure 2:**
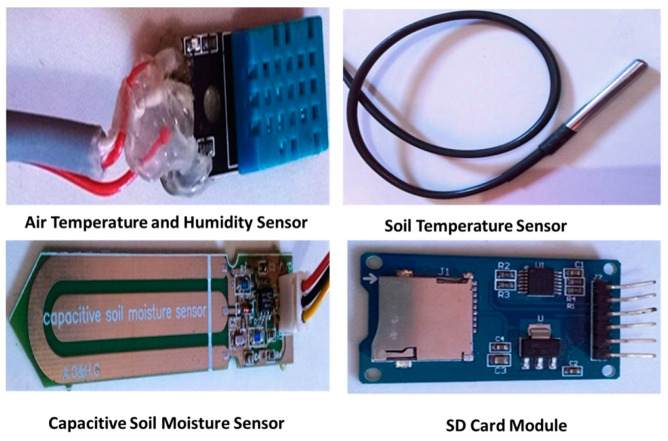
Different hardware components of the smart monitoring unit.

The sensor probe consists of a conductive copper plate located at the center, and a ground plate goes around it. The sensor probe consists of two parallel copper layers on a printed circuit board (PCB) FR-4 type double sided with 1.6 mm thickness, and the thickness of the copper is 35 μm. The dimensions of the effective part of the sensor were 20 mm by 60 mm, with a tipped end to insert it easily into the soil. The electronic tracking of the sensor was installed on the same PCB. The sensor electric circuit is basically an *RC* low-pass filter that receives a 430 kHz fixed square wave frequency from the 555 timer. Different parts of the electrical circuit of the proposed sensor are shown in Figure 1 and Figure 2. If the capacitance of the *RC* capacitor increases, the output signal will be filtered. Hence, the triangular signal will decrease in amplitude as the capacitance of C9 increases. A capacitor basically consists of conductive plates and a dielectric, and thus the capacitance of the capacitor is a function of the plates area and the permittivity of the dielectric between the plates or in the electric field around the plates. Thus, this capacitance can be changed by either changing the geometry of the copper plates or spacing between them. In our case, the geometry of the probe and the space between the parallel copper tracks are fixed; therefore, the change in the capacitance will be due to the change in the dielectric, according to Fares and Polyakov [31]:

(6) $$C\;=\;\varepsilon \frac{A}{D}$$

where ε is the dielectric constant (ε = εr ε_o_), εr is the dielectric constant of the medium, ε_o_ is the dielectric constant of the vacuum (ε_o_ = 8.854 pF m^−1^), *A* is the area of the conductive plate, and *D* is the separated distance.

However, theoretically, the variation in readings of the capacitive soil moisture sensor should change by factor of 80 in air or dry conditions compared with in water and wet conditions, but actually this does not occur as a result of the contribution of the sensor longitudinal geometry and insulating material coating the probe. The capacitance is calculated through [47]

(7) $$C\;=\;Ć\;+\;g\;\varepsilon_{o}\varepsilon b\;$$

where Ć is parasitic capacitance from the circuit connections; *g* is a geometrical factor in length units that mainly depends on the shape, dimensions, and separation of the sensor prongs; and εb is the soil apparent dielectric permittivity. The parasitic capacitance from the circuit connections (Ć) also contributes to the produced capacitance of the sensor.

### 2.2. Manufacture Stage

The sensor was manufactured in a workshop of the Benha El Harby factory for electronic industries. The probe of the sensor was designed on the P-CAD program, which is a commonly utilized program for PCB design. During the manufacturing process, the sensor probe was electrically insulated by coating it with a solder mask. The edges of the sensor were insulated by epoxy material. The sensor mask saves the probe from corrosion and also prevents/reduces interference from saline water. The designed sensor was tested in the laboratory by conducting two experiments. The first experiment included the simulation of the probe by using values for ceramic capacitors in the same range of the moisture from dryness to wetness (27 pF–460 pF), whereas the second experiment involved testing two different coating materials for insulting the sensor probe. Testing the materials was in both air and water (fresh and saline water).

### 2.3. Calibration Stage

#### 2.3.1. Soil Sample Preparation

Soil samples were collected from the experimental site, and then the samples were air-dried naturally. The main target of this step was to remove undesired materials such as rocks, stones, and roots, as well as to break down any large clumps of soil. Different chemical and hydro-physical properties of the experimental soil are listed in Table 1 and Table 2. The samples were then oven-dried at 105 °C for 24 h and then moisturized to have a range of soil water content by modifying the soil volumetric moisture content gravimetrically. The prepared-moisturized soil samples were transferred into plastic containers of one liter size. To avoid losing moisture during the calibration process, the containers were enclosed with a plastic cover, and the samples were protected after sealing for equilibrium. The sensors were inserted vertically to 6 cm depth into the core of the soil sample. Prior to taking the soil moisture measurements, the samples were left for 20 min to attain equilibrium [48].

#### 2.3.2. Sensors Variation Test

Before initiating the calibration procedure, the sensor-to-sensor measurement for the four tested sensors was preestablished and evaluated for soil content. The test was performed on clay textured soil. Soil samples of 40% water content were used for the test. The soil moisture measures were repeated 25 times for each soil sample. The noise of one sensor and the variability between the four sensors were analyzed by a one-way analysis of variance (ANOVA).

#### 2.3.3. Coefficient of Variation for Sensors

For the purpose of estimating the efficiency of the investigated sensors, the coefficient of variation (CV) was verified. Twenty-five replications of sensor measurements for every sensor were taken in samples having 30% moisture content. The CV was calculated for estimating the performance of the capacitive soil moisture sensor.

(8) $$\mathrm{CV}=\frac{\mathrm{SD}}{\chi^{\prime }}\times 100$$

where $\chi^{\prime }$ represents the mean of the sensor readings, and SD represents the deviations of the values from their arithmetic mean.

#### 2.3.4. Sensor Calibration

A created range of moisture content (0, 10, 20, 30, 40, and 50%) was used to calibrate the designed sensor and obtain a calibration function; the soil water content in the soil samples with varying moisture content was measured repeatedly using the sensor. During the calibration process, the sensor was inserted into the sample at 6 cm depth, and moisture readings were recorded, after which 100 g samples from each container were taken, weighed, and then directly oven dried at 105 °C for 24 h. For the lab experiments an Arduino Uno-developed board was used, and data were sent by USB cable-based serial port connection to be logged on a PC. After drying the soil samples, the volumetric moisture content was calculated through the following equation [49]:

(9) θv = θm × ρb

where *θv* is the volumetric soil moisture content, cm^3^·cm^−3^, *θm* is the gravimetric soil water content, g·g^−1^, and *ρb* is the soil bulk density, g·cm^−3^.

(10) $$\theta m\;=\;\frac{W\omega }{Ws}\times 100$$

where Wω is the mass of moisture content in the soil, and Ws is the mass of the dry soil.

### 2.4. Smart Monitoring Unit Design (Hardware and Software Description)

Following the lab tests and calibration of the sensor, the sensor was connected to a smart monitoring unit. The smart unit mainly comprised two parts. The first part consisted of a group of sensors: a capacitive soil moisture sensor, soil temperature sensor, and air humidity and temperature sensor, which were used to monitor various environmental parameters inside the greenhouse. All the collected data were directly sent to an SD memory card. A microcontroller was responsible for controlling these sensors. Figure 2 shows the different sensors of the smart monitoring unit. The second part was a powering unit (solar panel, battery, and charger), which operated the system.

#### 2.4.1. Microcontroller

A microcontroller is basically the main brain of any smart monitoring system. Here, it was an 8-bit AVR that was a member of the ATmega 328p family from ATEMEL. This microcontroller had (combined) EEPROM-1 KB, SRAM-2KB, flash memory-32 KB-23 programmable I/O lines.

#### 2.4.2. Soil Temperature Sensor

A waterproof DS18B20 (Dallas Semiconductor, Dallas, TX, USA) sensor was used to measure the soil temperature (Figure 1). This sensor can measure a range from −55 °C to 125 °C with an accuracy of 0.5 °C. The soil temperature sensor was connected to the microcontroller through three pins. The Vcc pin of the DS16B20 sensor connected to +5 V of the microcontroller, the GND pin of the sensor connected to the GND of the microcontroller, data pin connected to D4 on the microcontroller, and the Vcc pin was linked to a data pin via 4.7 kΩ as a pull-up resistor. The communication of this sensor with the microcontroller was through one wire protocol.

#### 2.4.3. Air Relative Humidity and Temperature Sensor

The relative humidity and the temperature of the air inside the greenhouse were sensed using a DHT11 sensor. It operated at a volt range of 3.3 V to 5 V (analogue output). The humidity measuring range of the sensor ranged between 20% and 90%, with ±5% accuracy, while the temperature measuring range was 0 °C to 50 °C, with an accuracy of ±2 °C. The DHT11 analogue sensor module consisted of three pins: two of them for the power (GND and Vcc), which was connected to the power pins of the microcontroller, and a third data pin was connected to A0 (analog to digital converter) on the microcontroller (Figure 2). The humidity and temperature sensor were installed at 2 m height from soil surface.

#### 2.4.4. SD Card Module

During the field experiment, the collected data were sent to an SD memory card. The SD module consisted of 6 pins: two pins for powering the module and four pins for serial peripheral interface (SPI). The pin 1 connected to the GND of the microcontroller, pin 2 connected to the +5 V microcontroller, pin3 (MISO) connected to D12, pin4 (MOSI) connected to D11, pin5 (SCK) connected to D13, and pin6 (CS) connected to D10 of the microcontroller. The hardware components used in the monitoring unit are shown in Figure 2. The total cost of the used monitoring unit was about USD 70.00.

### 2.5. Powering Unit

**Figure 3:**
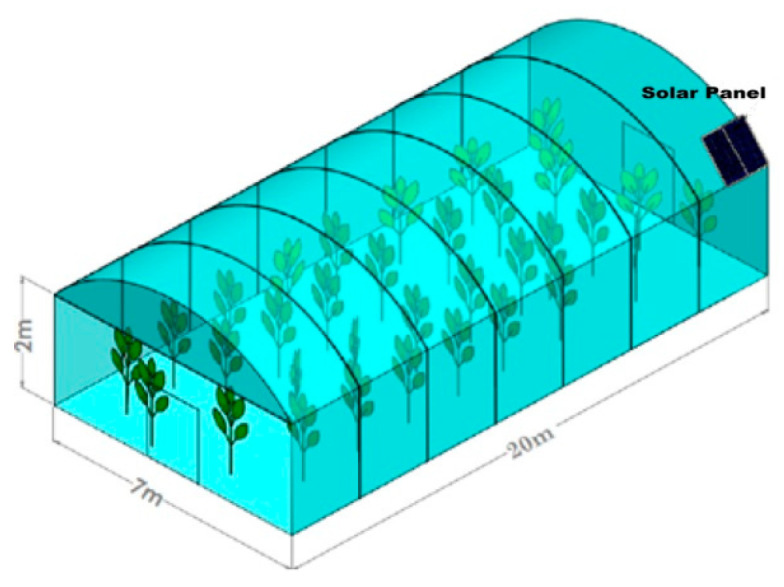
A schematic diagram of the greenhouse showing dimensions and the position of the solar panel on the roof of the greenhouse.

The powering unit included photovoltaic panels of 120 W–22 V–6 A placed on the roof of the greenhouse surface (Figure 3) where sunlight intensity was expected to be higher. These solar panels, during the daytime, charged the batteries that operated the control unit. The rest of this unit comprised a charger (12 V/24 V–30 A) and MPPT unit, which maximized the power rate under different conditions, and a 12 V–7 A battery.

*Isc* was measured in series, and *Voc* was measured in parallel via a multimeter device, and the vertical irradiance was also measured. Input and output panel surface temperature, air relative humidity, and temperature were also measured. During the calibration, the solar panel was oriented south with a 30° tilt angle. The testing started from 07:30 to 18:00. The input and output power was calculated by the formulae of Lal et al. [50]:

(11) Pout = Voc × Isc

where *Pout* is the output power (W), *Voc* is the open circuit voltage (volt), and *Isc* is the short circuit current (*A*). The input power was calculated as follows:

(12) Pin = Ins × A

where *Pin* is the input power (W), *Ins* is the irradiance (Wt/m^2^), and *A* is the area of the solar module (m^2^). The efficiency of the panel is given by Lal et al. [50]:

(13) $$ȵ\;panel\;=\;\frac{Pout}{Pin}\times ff$$

where ȵ panel is PV *panel* efficiency (%), *Pout* is output power (W), *Pin* is input power (W), and *ff* is fill factor.

### 2.6. Scenario 2 (Field Stage)

#### 2.6.1. Site Description

The experimental work was carried out during spring season of 2019 at the Research Farm, Sakha, Kafresheikh province (latitude of 31°05′45.34″ N and longitude 30° 56′ 41.40″ E), with an elevation of about 7 m over the mean sea level. This region is characterized by a dry climate with mean annual temperature of 20 °C and rain fall of 29 mm. The direct solar irradiation intensity ranges between 2000 to 3200 Wh/m^2^/day, and the sunshine hours range between 9 to 11 h/day, with very few cloudy days over this period of time [51]. Physical and chemical properties of the experimental soil are listed in Table 1 and Table 2. The manufactured soil moisture sensor and smart monitoring system was experimentally tested under field conditions to evaluate the efficiency of the system for detecting the irrigation occurrences and real time monitoring of moisture across the root zone.

The smart monitoring unit was installed in the greenhouse, which was cultivated with cucumber (fifa sun). Cucumber plants were transplanted in beds of 1.0 m width. The bed had two rows of plants, with 0.50 m plant spacing. Two laterals of 0.50 m emitter spacing were allocated, one for each crop row. The emitters of 2.64 L·h^−1^ discharge rates were used to meet the water requirements of the plants. The capacitive soil moisture sensors were vertically installed at 0.20 m depth to the center of probe and at 0.15 m away from the emitter. To automatically record soil moisture measurements, different soil moisture sensors were attached to the microcontroller. Every sensor had three output sockets: the first was connected to the power pin, the second and the third were connected to analogue converter and ground pins of the microcontroller. The soil moisture records were saved on the SD card, which could be uploaded to a PC. Irrigation requirements were based on the International Center for Agricultural Research in Dry Areas (ICARDA) [52] and were calculated to evaluate the adopted irrigation approach in the farm and to detect irrigation episodes. The evapotranspiration was calculated from the following equation:

(14) $$ETc\;=\;ET_{o}\;\times \;Kc$$

where *ETc* is the total evapotranspiration (mm/day), $ET_{o}$ is the standard evapotranspiration (mm/day), and *Kc* is the crop factor. The actual amount of water added was measured volumetrically during the irrigation process.

#### 2.6.2. Infiltration Rate

The infiltration rate was identified using a double ring infiltrometer with standard dimensions. Care was taken to ensure that the double rings were buried vertically into the soil to 15 cm depth, then the inner ring and the space between both rings was filled with water to 10 cm height. Maintaining water to a constant head, the infiltrated volume through the time in the inner ring was applied to the previous head using a measuring cylinder and a stopwatch. This action was repeated to achieve a saturated condition and stabilization of water infiltration with the time. The saturated hydraulic conductivity was calculated according to [24]:

(15) $$Ksat\;=\;\frac{∆v}{∆t\;\times \;A}$$

where Ksat is the saturated hydraulic conductivity (mm/h), ∆*v* is the change of water volume in the inner ring (m^3^), ∆*t* is the time between the changes of the volume (s), and *A* is the inner ring area (m^2^).

#### 2.6.3. Emitter Evaluation

The performance of the drip irrigation network in the greenhouse was evaluated prior to conducting the experiment. The network had lateral lines of 16 mm diameter equipped with the emitters of 50 cm spacing, from which four lateral lines were checked at 1 bar operational pressure. The emitters were selected from the first, second, third, and fourth quarter from each line, where the measurement was taken for every two emitters in the quadrant to calculate the mean. The discharge was collected in cans for five minutes, and then the collected amount of water in each catch was transferred to a graduated cylinder to determine the volume. The evaluation of the drip system was used to determine application efficiency and emission uniformity. The uniformity of application was estimated with the Christiansen uniformity coefficient according to ASAE [53]. The coefficient of manufacturer’s variation was determined according to James [22]. Emission uniformity was calculated according to Burt et al. [54].

### 2.7. Statistical Analysis

Following several established research studies reported in the literature, the current research analysis was assessed statistically using the root-mean-square error (RMSE) metric. The standard deviation of the residuals was calculated to observe the variation in soil moisture sensors’ response measurements at 30% moisture content. The residuals provide a measure of how closely the observations fit the regression line. The RMSE metric was used to verify the experimental findings that were established in the current study following the research by Sharma et al. [55] reported in the literature. On the other hand, the least significant difference (LSD) test was calculated after the analysis of variance when the *F*-ratio suggests the rejection of the null hypothesis H0—that is, when the difference between the population showed no obvious change. The main idea of the LSD test is to evaluate the populations taken in pairs. It is then used to run in a one-way or two-way analysis of variance, considering that the null hypothesis has already been rejected [56], and the LSD test was calculated as follows:

(16) $$LSD\;=\;t\alpha \sqrt{\frac{2\;MS\;E}{n}}$$

where *MS E* is the standard error of differences between two means, *n* is the number of observations and *tα* is the *t* is the *t* value at 0.05 probability level.

## 3. Results and Discussion

### 3.1. Laboratory Results

#### 3.1.1. Results of Test Board Experiment

Table 3 shows the calculated cut-off frequency according to Equation (5) compared with the actual values of the cut of frequency that was tested and measured by oscilloscope device. When the capacitive probe was inserted into a dry soil, its capacitance was low (27 pF). Therefore, the cut-off frequency of the low-pass filter was about 589.46 kHz, and it was expected to be just over 3 dB signal attenuation. However, when the probe was inserted into a wet soil, the sensor capacitance rose to 300 pF upwards, which is associated with lower cut of frequency of 53.05 kHz downwards. It should be noted that a square wave was used in this case, whereas the theoretical 3 dB attenuation “corner frequency” applies only on sine wave signals, which means there is a significant component harmony of the base frequency at odd multiples. Additionally, these harmonics are ordinarily above the corner frequency of the low-pass filter, so there is no issue affecting sensor performance.

#### 3.1.2. Coating Material Effects on Sensor’s Performance

**Figure 4:**
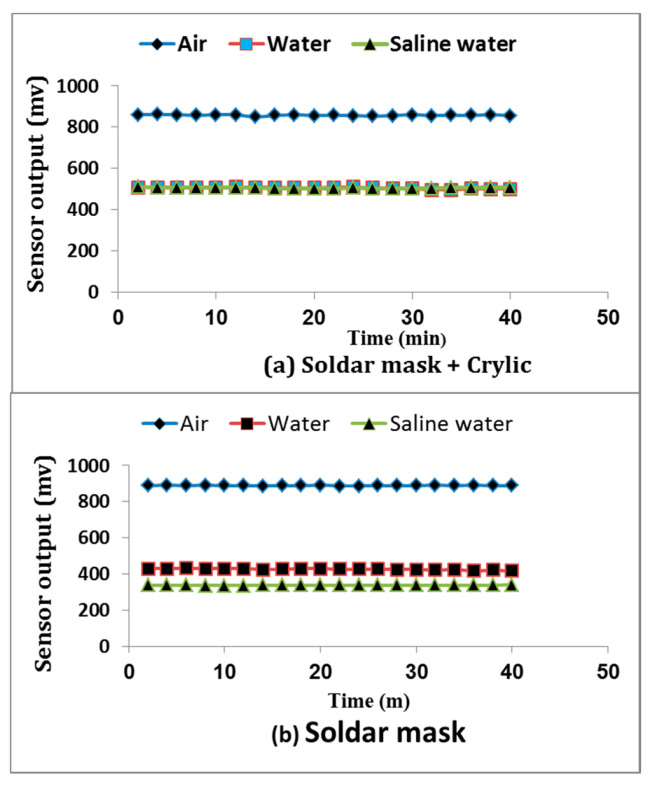
Sensitivity response of the designed sensor with both tested coating materials: (**a**) solder mask + acrylic; (**b**) solder mask measured in air, fresh water, and saline water.

Figure 4 shows the sensor sensitivity response in readings when coated by solder mask and when coated by solder mask plus acrylic varnish in varying conditions (air, water, and saline water). It is obvious that solder mask with acrylic had a stable response in both fresh and saline water (salinity up to 20 dS m^−1^). However, the solder mask had response changes for saline water. Thus, the sensor with acrylic varnish is unsusceptible to the ions of the salt (no direct current), and thus one of the problems of measuring soil moisture content based on the dielectric technique was managed. Additionally, there were no significant differences in the readings of the sensor insulated by solder mask and cosmetic acrylic varnish either in fresh or saline water.

#### 3.1.3. Sensor’s Variability

The ANOVA analysis results for the different tested capacitive soil moisture sensors with 25 replications are listed in Table 4. As can be seen, the variability is divided into two categories: (i) variability between the sensors (sensor-to-sensor variability) and (ii) variability of the sensor response measurements and replications (precision). The results demonstrated that the *F* value is greater than the critical *F* value, which means there are significant differences in the mean values of sensor response. Subsequently, the null hypothesis on which our research was based was rejected and the alternative hypothesis was accepted, allowing us to assert that the mean between sensors was not equal. The ANOVA results showed significant differences when comparing between the four tested sensors in pairs or as a group. Therefore, the LSD test was performed demonstrating significant differences between the mean of S1 and the other three (S2, S3, S4), while there were no significant differences between the means of S2, S3, and S4 (the differences between the means were <1.697).

#### 3.1.4. Coefficient of Variation for the Sensors

As shown in Table 5, although the coefficient of variation (CV) of the four tested sensors is not equal but are less than 25%, it means that the performance of all sensors is high for estimating the soil moisture content at a range of the field capacity of clay-textured soils. The first sensor was the optimum for measuring soil moisture content at 30% with a CV value around 0.02%, while the fourth sensor performed less well with a CV of 0.09%.

#### 3.1.5. Calibration of Capacitive Soil Moisture Sensor

**Figure 5:**
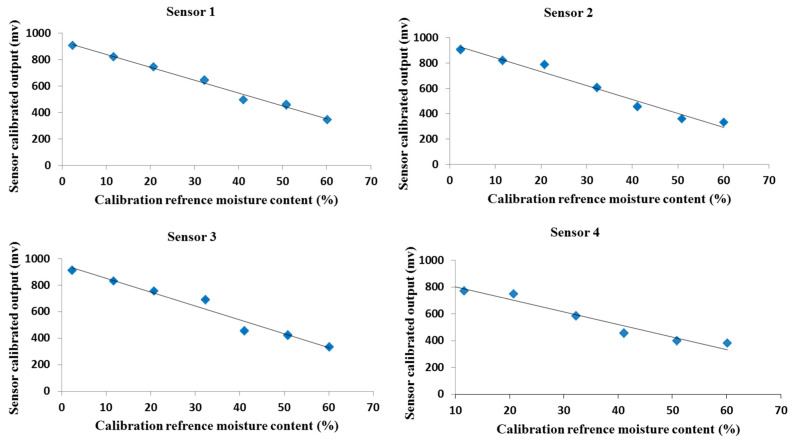
Calibration results of different soil moisture sensors tested. The results of the analysis are presented in Table 6.

As presented in Figure 5, the measured soil moisture sensor output declined gradually with increased soil moisture content. Based on the method of least squares, the measured millivolt by the designed sensor correlated significantly with the soil moisture content with *R*^2^ value for various sensors of 0.989, 0.972, 0.966, and 0.971 for S1, S2, S3, and S4, respectively, with a linear equation as a calibration function. From these simple linear relationships, the values of *R*^2^ between the volumetric moisture content (*θ_V_*) and the millivolt produced from the sensors were greater than 0.95 for all tested sensors. Hence, this correlation suggests that the designed sensor is favorably adaptable for measuring soil moisture content. Although the sensor-to-sensor variability test demonstrated variations between the sensors under the same conditions, the specific calibration for every sensor showed a strong correlation between *θ_V_* and sensor millivolt. Earlier studies have been implemented to develop sensors to estimate soil moisture content. Among these techniques, the dielectric technique is more advantageous for its automation ability and on site records of moisture, in addition to its great accuracy and wider range of θ_V_ [57]. The defect of using dielectric methods to estimate moisture content is that the relationship between *θ_V_* and soil permittivity mainly depends on soil texture [58], and thus a certain calibration setup is required for every soil type [59]. Regression analysis showed a high correlation between *θ_V_* and millivolt produced by the capacitive soil moisture sensor, and the lowest values of the RMSE were achieved for every sensor. The maximum *R*^2^ and adjusted *R*^2^ were 0.989 and 0.987, respectively recorded by sensor 1 with RMSE of 0.018, while the minimum *R*^2^ and adjusted *R*^2^ were 0.965 and 0.957, respectively recorded by sensor 3 with RMSE of 0.012. Both sensor 3 and sensor 4 have roughly the same *R*^2^ and adjusted *R*^2^ (see Table 6). The results demonstrate a direct relationship between the actual values of *θ_V_* and the predicted values that are based on linear regression equations for every sensor. Moreover, the predicted *θ_V_* gradually increased as the actual values of moisture content increased with *R*^2^ values of 0.952, 0.966, 0.951, and 0.972 for S1, S2, S3, and S4, respectively. It is also obvious that sensor 4 had the optimum performance for the prediction of *θ_V_*. With these great efficiencies of different sensors for measuring *θ_V_*, this soil moisture sensor can be a reliable tool in irrigation scheduling. When these sensors are attached to a smart monitoring irrigation unit, they would save the amount of water required for irrigation and thus increase water use efficiency. As reported in previous studies, accurate sensors should have RMSE values of zero or nearly zero, which means no difference between sensor readings and real values [46].

### 3.2. Results Obtained from the Greenhouse

#### 3.2.1. Solar Panel Calibration

**Figure 6:**
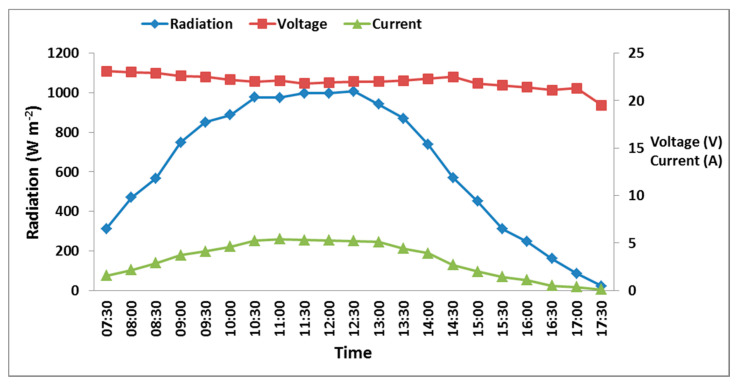
Measured irradiance and both voltage and current produced from the solar panel over daytime.

As depicted in Figure 6, the peak of solar radiation was recorded from 09:00 to 14:00, with an average value of 743 W/m^2^. The current generated from the tested solar panel was highly related with the change in the radiation that was recorded—1.54 A at 07:30 at radiation value of 312.60 W/m^2^—and both parameters increased with the day’s passing time until they reached their peak at 10:30 and remained relatively constant until 12:30. After that, the radiation and the current started to decrease until recording their minimum values at 17:30. On the contrary, the change in voltage was little throughout the day, and the highest value of 23.1 V was recorded at 07:30, while the lowest value of 19.5 V was recorded at 17:30. The greatest output was recorded between 09:00 and 14:00, which is close to 80–85% of the 120 W output expected from the solar cells. Although it is expected that the maximum output power should be at 12:00 due to the peak of the radiation and because the input power is highest, the produced power decreased because of the increasing temperature of the solar panel surface. The calibration test also showed that the maximum solar panel efficiency was 11.08% at 11:00, with 945.40 W/m^2^ and 55.14 °C for the sun radiation and the surface temperature of the solar panel, respectively.

#### 3.2.2. Implementation of the Monitoring Unit

**Figure 7:**
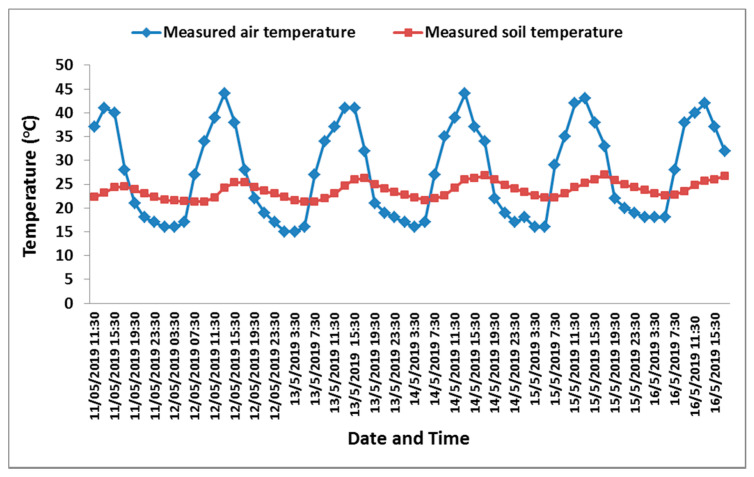
Air and soil temperature (°C) recorded by the designed automated monitoring system.

**Figure 8:**
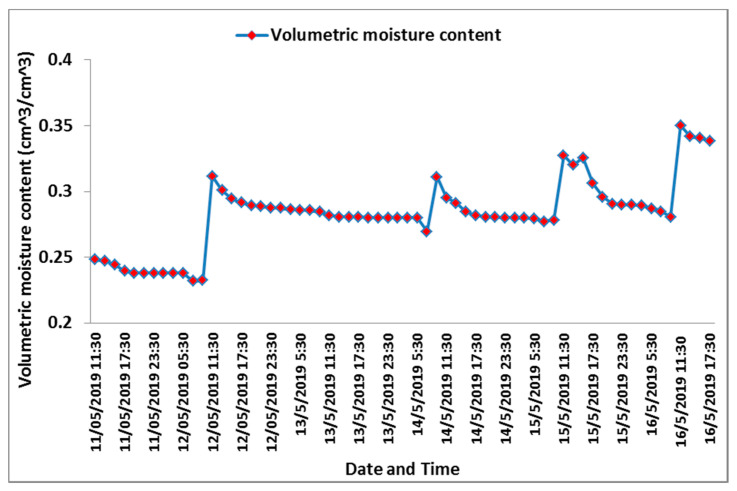
Volumetric moisture content (*θ_V_*) recorded by the designed automated monitoring system.

Following the assessment of the response of the designed sensor in the lab, it was also tested under actual field conditions to evaluate its performance in a real-time condition (inside controlled greenhouses). The sensors were evaluated in a greenhouse cultivated with cucumber as a vegetable crop. The sensors were adjusted to use the calibration curve of the clay soil for estimating *θ_V_* from voltage. The smart monitoring unit detected irrigation events that occurred over the experimental period. The first irrigation event happened on 11 May at 09:47, the second irrigation occurred on 12 May at 09:11, the third irrigation took place on 14May at 08:50, while the fourth event on 15 May at 09:22, after which the fifth irrigation occurred on 16 May at 09:38. The measurements of air temperature and soil temperature recorded by the smart monitoring unit are depicted in (Figure 7) during the test period. Soil temperature began to rise from 11:30, with an average temperature of 23.46 °C, continuing onwards until reaching the maximum of 26.1 °C at 17:30. However, compared with the change in air temperature, the change in soil temperature throughout the day was slight due to the effect of mulching. During the hours of brightest sun, the air temperature increased until reaching the maximum at 13:30, and then the temperature began to decrease. The values of the measured *θ_V_* increased after irrigation actions. The smart monitoring unit measured and recorded the volumetric water content (*θ_V_*), which ranged between 0.23 and 0.35 m^3^ m^−3^, as illustrated in Figure 8. During the experiment, the total applied amount of irrigation water was 27.55 mm, and the amount of irrigation water detected by the experimental sensor was 26.45 mm, which was calculated by taking the average efficiency of the four sensors.

**Figure 9:**
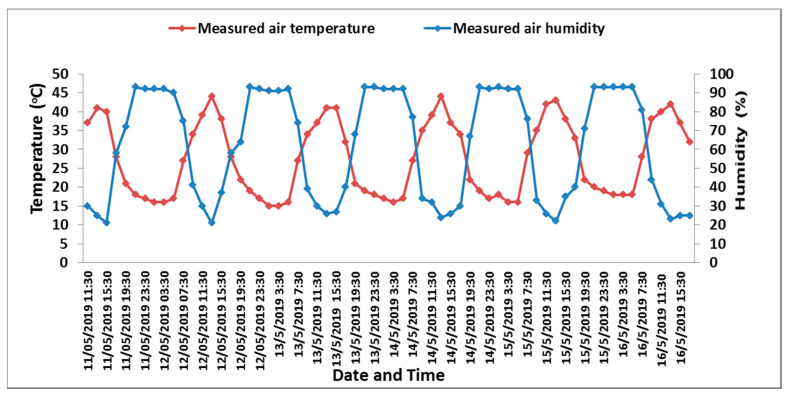
Air temperature (°C) and relative humidity (%) recorded by the designed automated monitoring system.

Regarding the air humidity and temperature recorded by the smart monitoring unit, a cyclic behavior is shown in Figure 9. The relative humidity of the air inside the greenhouse began to decrease with the sunshine until it reached its lowest value at 13:30. Additionally, a sudden increase in air relative humidity was observed at 19:30, reaching its maximum at 21:30, then remaining constant until 05:30, at which time it started to decrease associated with the sunshine. It is obvious that the smart monitoring system is reliable and can be utilized for greenhouse irrigation management.

The main obstacle of the designed soil moisture sensor (soil moisture sensor capacitance-based) is that it is affected by the soil temperature, as reported previously by Oates et al. [39]. In our research, the effects of the soil temperature were reduced due to mulching. In addition, ground noise of the sensor can be averted by supplying the sensor with a DC power supply. This can reduce the ripple and make the produced signal more stable. The designed capacitive soil moisture sensor can be employed successfully as a complementary component of automated irrigation system for irrigation water management in controlled greenhouses or even in the open field. It is an alternative low-cost, effective, and a reliable solution compared with expensive soil moisture commercial devices. With respect to the results obtained, more research is required to test and calibrate the designed sensor with different soils (e.g., loamy and sandy) to ensure its efficiency in irrigation scheduling.

#### 3.2.3. Drip Irrigation System Evaluation

The field assessment of a drip irrigation network is important for scheduling irrigation water by a reliable method. Coefficients of manufacturer’s variation (CV), Christiansen’s coefficient of uniformity (CU), distribution uniformity (DU), and flow rate variation (q_var_) at different emitter lines are shown in Table 7. The obtained data indicate that the performance of the drip irrigation system was good according to ASAE (1998a), where the mean values of CV, CU, DU, and q_var_ were 0.037, 99.05%, 95.55%, and 8.14%, respectively.

## 4. Conclusions

This research aimed to design and calibrate a low-cost, effective capacitive soil moisture sensor attached to a smart monitoring unit that could be used in controlled greenhouses. Lab experiments were conducted to demonstrate the designed soil moisture sensor and to evaluate its performance through its connection with a smart monitoring unit. A sensor-to-sensor variation test showed minor variations in response measurements of the four investigated sensors. In addition, the calibration results showed positive correlation between the volumetric moisture content and millivolt generated by the sensor, with determination coefficient values (*R*^2^) of 0.989, 0.972, 0.966, and 0.971 for sensors 1, 2, 3, and 4, respectively, all of which were evaluated under the same conditions. The results prove that the designed capacitive soil moisture sensor would be a robust effective instrument for measuring and monitoring volumetric moisture content with reliable readings in clay textured soils. The smart monitoring unit was used to monitor different environmental conditions inside the greenhouse, including the volumetric soil moisture content, soil temperature, air temperature, and humidity. The greenhouse results demonstrate that the smart monitoring system effectively detected irrigation events and other environmental conditions inside the greenhouse. During the field test, the data were sent to an SD memory card, and thus the system can be used for irrigation management in greenhouses and open fields. The scope of future studies is to develop the designed capacitive sensor and calibrate it with different types of soils, as well as to assess the performance of the sensor at a wider temperature range using a temperature-controlled chamber. Furthermore, the intention is to develop a smart unit that can be used as an automated irrigation system that employs deep learning to predict crop water requirements.

## Figures and Tables

**Table 1:** The physical characteristics of the studied soil experiments.

Field Sampling Depth, cm	Particle Size Distribution			FC, %	WP, %	*ρ_b_*, g cm^−3^	AW, %
	Sand	Silt	Clay				
0–15	13.0	32.5	54.50	39.50	19.50	1.25	20.00
15–30	13.70	32.0	54.30	40.10	20.50	1.26	19.60
30–45	13.90	33.00	53.10	40.00	20.40	1.29	19.60

FC: field capacity; WP: welting point; *ρ*b: bulk density; AW: available water.

**Table 2:** Some chemical properties of the experimental soil.

Field Sampling Depth, cm	pH	EC, dSm^−1^ dSm^−1^	Cations, meq/L				Anions, meq/L				SAR
			Na^+^	Ca^++^	Mg^++^	K^+^	Co_3_^−^	Hco_3_^−^	Cl^−^	So_4_^−^	
0–15	8.1	3.70	25.20	7.8	4.4	0.4	0.0	5.50	17.60	14.60	10.18
15–30	8.0	4.00	27.20	8.4	4.8	0.4	0.0	5.00	19.00	16.80	10.59
30–45	8.2	4.20	28.60	8.8	5.0	0.4	0.0	5.50	20.00	17.30	10.85

**Table 3:** Calculated and measured cut-off frequency of low-pass filter vs. ceramic capacitor’s simulation of the sensor probe.

Capacitance, pF	Calculated Cut off Frequency, kHz	Measured Cut off Frequency, kHz
27	589.46	589.21
47	338.62	338.57
100	159.15	159.15
140	113.68	113.60
174	91.46	91.35
210	75.78	75.69
350	45.47	45.23
460	34.59	34.50

**Table 4:** ANOVA results for the 25 replications of the response measurements for different tested moisture sensors at 40% soil moisture content.

Sensor	Observations	Sum	Average	Variance				
S1	25	12,334	493.4	1.16				
S2	25	11,389	455.6	2.01				
S3	25	11,385	455.4	2.42				
S4	25	11,375	455.0	1.08				
**ANOVA**								
**Source of Variation**	**SS**	**df**	**MS**	***F***	***p*** **-Value**	**F_crit_**		
Between groups	27,136	3	9045.39	5429.95	5.62 × 10^−107^	3.99		
Within groups	159.9	96	1.666					
Total	27,296	99						
**LSD**								
**tα**	**MS**	**LSD**	**S_1_–S_2_**	**S_1_–S_3_**	**S_1_–S_4_**	**S_2_–S_3_**	**S_2_–S_4_**	**S_3_–S_4_**
2.66	1.66	1.69	37.8	37.96	38.36	0.16	0.56	0.4

**Table 5:** Coefficient of variation (CV), standard deviation (SD) and mean response (MR) of the designed soil moisture sensors response measurements at 30% moisture content.

Sensor No	SD	MR	CV
S1	0.137	643.63	0.02
S2	0.230	603.90	0.03
S3	0.278	684.45	0.04
S4	0.520	587.95	0.09

**Table 6:** Regression analysis results: equation, determination coefficient (*R*^2^), root-mean-square error (RMSE) between sensor readings (millivolt) and gravimetric moisture content.

Sensor No.	Equation	*R* ^2^	Adj *R*^2^	SE	RMSE
S1	y = −9.7565x + 935.22	0.989	0.986	0.0031	0.018
S2	y = −10.971x + 952.44	0.971	0.966	0.0049	0.009
S3	y = −10.467x + 955.45	0.965	0.957	0.0055	0.012
S4	y = −9.3154x + 893.57	0.964	0.958	0.0054	0.019

**Table 7:** Drip irrigation network evaluation calculating coefficient of variation (CV), Christiansen’s coefficient of uniformity (CU), distribution uniformity (DU), and flow rate variation (q_var_) at 1 bar operating pressure.

Lateral Line	CV	CU, %	DU, %	q_var_, %
1	0.038	98.43	96.67	6.45
2	0.040	98.23	95.25	9.15
3	0.029	99.13	95.67	7.72
4	0.041	99.55	94.62	9.27
Mean	0.037	98.83	95.55	8.14

## Data Availability

The data presented in this study are fully available in this article.
